# Cross-talk between chronic lymphocytic leukemia (CLL) tumor B cells and mesenchymal stromal cells (MSCs): implications for neoplastic cell survival

**DOI:** 10.18632/oncotarget.6239

**Published:** 2015-10-26

**Authors:** Valentina Trimarco, Elisa Ave, Monica Facco, Giorgia Chiodin, Federica Frezzato, Veronica Martini, Cristina Gattazzo, Federica Lessi, Carlo Alberto Giorgi, Andrea Visentin, Monica Castelli, Filippo Severin, Renato Zambello, Francesco Piazza, Gianpietro Semenzato, Livio Trentin

**Affiliations:** ^1^ Padua University School of Medicine, Department of Medicine, Hematology and Clinical Immunology Branch, Padua, Italy; ^2^ Venetian Institute of Molecular Medicine (VIMM), Padua, Italy

**Keywords:** chronic lymphocytic leukemia, mesenchymal stromal cells, kinase inhibitors

## Abstract

Leukemic cells from Chronic Lymphocytic Leukemia (CLL) patients interact with stromal cells of the surrounding microenvironment. Mesenchymal Stromal Cells (MSCs) represent the main population in CLL marrow stroma, which may play a key role for disease support and progression. In this study we evaluated whether MSCs influence *in vitro* CLL cell survival. MSCs were isolated from the bone marrow of 46 CLL patients and were characterized by flow cytometry analysis. Following co-culture of MSCs and leukemic B cells, we demonstrated that MSCs were able to improve leukemic B cell viability, this latter being differently dependent from the signals coming from MSCs. In addition, we found that the co-culture of MSCs with leukemic B cells induced an increased production of IL-8, CCL4, CCL11, and CXCL10 chemokines.

As far as drug resistance is concerned, MSCs counteract the cytotoxic effect of Fludarabine/Cyclophosphamide administration *in vivo*, whereas they do not protect CLL cells from the apoptosis induced by the kinase inhibitors Bafetinib and Ibrutinib. The evidence that leukemic clones are conditioned by environmental stimuli suggest new putative targets for therapy in CLL patients.

## INTRODUCTION

The accumulation of CD19+/CD5+/CD23+ B cells is a peculiar hallmark of B cell chronic lymphocytic leukemia (CLL) [1]. Despite a remarkable phenotypic and cytological homogeneity, CLL is characterized by extremely variable clinical course related to different prognostic factors including the mutational status of the immunoglobulin heavy-chain variable region (IgV_H_) [2-4], expression of CD38 and ZAP70 markers [5, 6] and specific cytogenetic alterations [7-9]. Although leukemic cells show prolonged lifespan in the peripheral blood, secondary lymphoid organs and bone marrow (BM), they rapidly undergo spontaneous apoptosis *in vitro* [10, 11], suggesting that the leukemic B cells survival advantage could be attributed not only to intrinsic defects of apoptotic mechanisms but also to signals delivered by accessory cells at the sites of the disease activity. In tissue microenvironment, CLL B cells reside in close contact with T lymphocytes, stromal cells, mesenchymal stromal cells (MSCs), endothelial cells, follicular dendritic cells and macrophages. Interactions among these components of the microenvironment regulate the trafficking, survival, and proliferation of leukemic B cells in a way that depends both on direct cell-cell contact and/or on the exchange of soluble factors [12]. Moreover, once resident in stromal environment, CLL cells are protected from different therapeutic interventions [13-15].

Among bone marrow stromal cells, MSCs show a bidirectional cross-talking with neoplastic B cells. Leukemic cells are supported by stromal cells and, in turn, are also able to activate and induce stromal cell to proliferate and release several mediators (i.e., CXCL12, CXCL13, CCL19 and CCL21) which sustain the ongoing process [16-18]. These interactions drive CLL B cells into tissue microenvironment, where malignant cells experience the survival and proliferation signals mediated by the B cell receptor (BCR) and other pathways [15]. Nevertheless, these complex cellular and molecular mechanisms are not yet completely defined. Although in healthy subjects MSCs represent a small fraction of the stromal cell population, immunohistochemistry studies performed in patients with several lymphoproliferative diseases showed that *in situ* αSMA^+^ mesenchymal stromal cells, which represent the *in vivo* counterpart of MSCs, are the dominant stromal cell population in CLL microenvironment [19]. These observations support a crucial role of MSCs on the mechanisms favoring malignant cells and disease progression in CLL.

In the last years, the modulation of tumor microenvironment is becoming a promising therapeutic strategy in CLL treatment, demonstrated by the use of an increased number of compounds (i.e. thalidomide, lenalidomide, plerixafor and natalizumab) [20, 21], affecting molecules involved in the compartimentalization of tumor cells. More recently, several small molecules have been developed to inhibit a variety of kinases in the BCR pathway, including Lyn, Syk, Btk and PI3K, which are crucial not only for the activation of multiple survival pathways (such as Akt, Erk, NF-kB) but also for chemokine-mediated migration and adhesion of B cells in the microenvironment [22]. Thus, the understanding of the interactions between CLL B cells and the microenvironment is mandatory to define more effective therapies for CLL. In this context, the main aim of this study was to investigate the *in vitro* impact of MSCs on CLL B cell survival in order to verify whether MSCs protect leukemic B cells from spontaneous apoptosis both at basal conditions and after *in vivo* Fludarabine and Cyclophosphamide containing regimen therapy. We also tested the effect of two kinase inhibitors, Bafetinib (dual BCR-Abl/Lyn inhibitor) and Ibrutinib (Btk inhibitor), known to reduce neoplastic B cell viability *in vitro* [23], on CLL B cells in presence of MSCs. Moreover, the investigation of soluble factors, mainly cytokines and chemokines, which could be involved in leukemic cell survival, was performed. Our data clearly demonstrated that MSCs display a pro-survival effect on leukemic B cells from CLL patients and that CLL clones displayed a variable degree of responsiveness to microenviromental stimuli, suggesting that same clones are dependent and other are independent from MSC pro-survival capability. This observation might be relevant in order to identify patients who may benefit of compounds targeting CLL microenvironment.

## RESULTS

### Mesenchymal stromal cells from CLL patients display phenotypic profile and differentiation capability of MSCs from normal subjects

MSCs were obtained from the bone marrow of 46 CLL patients by plastic adhesion as previously described [24, 25]. The adherent fraction leads to the formation of high proliferating spindle-shaped colonies, reaching the confluence in 30 days (Figure S1A). Flow cytometry analysis showed that MSCs were positive for CD90, CD73, CD105, and negative for CD14, CD34, CD45 and CD31 (Figure S1B). MSC ability to differentiate in adipocytes and osteocytes was tested using specific conditioned media. Adipogenic differentiation was demonstrated by the detection of lipid vesicles in the cytoplasm of (pre)adipocytes, stained with Oil Red. Osteogenic differentiation was documented by the increased expression of mRNA coding for Core Binding Factor 1 (CBFA1) and the deposition of mineralized matrix, shown by the Von Kossa staining (Figure S1C). The cell adhesion, the immunophenotype and the differentiation ability of stromal cells generated in our cultures are in accordance to the criteria required for MSC characterization [26, 27].

### MSCs from CLL patients support *in vitro* neoplastic B cell survival

**Figure 1:**
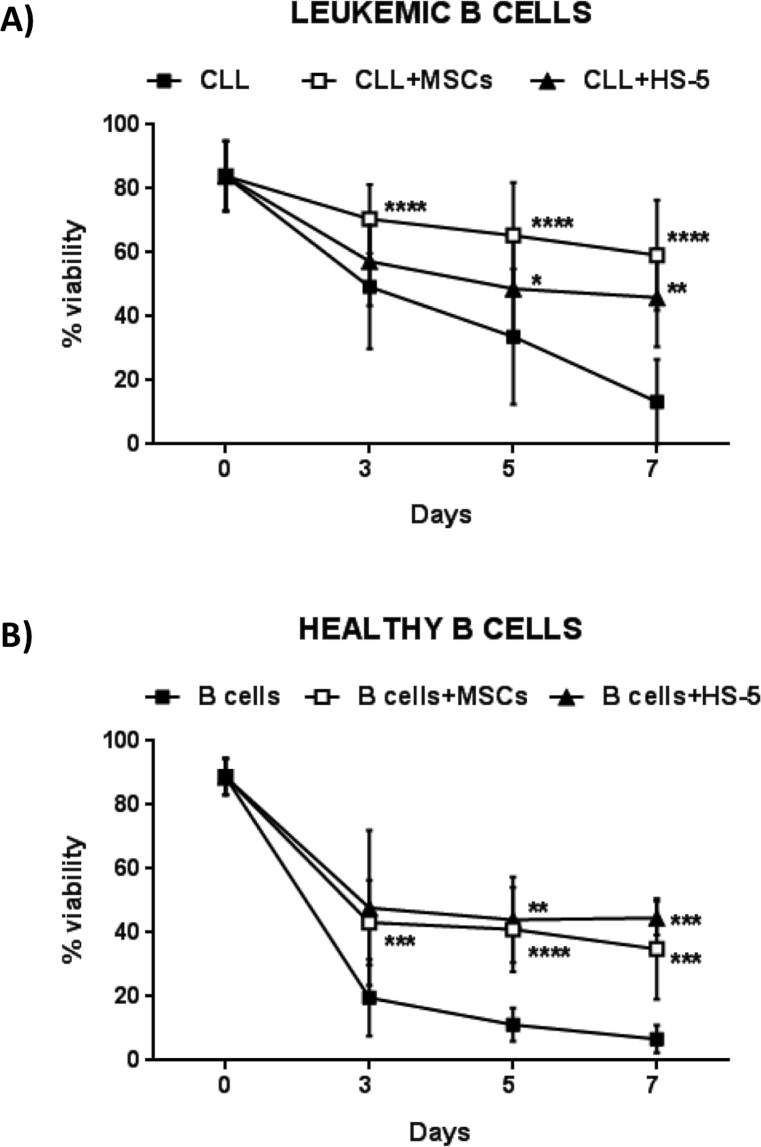
MSCs protect CLL B cells from spontaneous apoptosis **A.** Survival of CLL B cells cultured alone (*n* = 30), in presence of MSCs (*n* = 30) and of the stromal cell line HS-5 (*n* = 16). **B.** Survival of B cells from healthy donors cultured alone (*n* = 11), in presence of MSCs (*n* = 11) and HS-5 (*n* = 5). Viability was measured at the time point of 0, 3, 5 and 7 days by the Annexin V staining. Data are presented as mean ± standard deviation; paired Student's *t* test; *p < 0.05, **p < 0.01, ***p < 0.001 and ****p < 0.0001.

We tested the effect of MSCs on the survival of leukemic B cells obtained from 30 CLL patients and normal B cells from 11 healthy donors. The assays were performed incubating leukemic and normal B lymphocytes in contact with a confluent layer of MSCs (20:1 ratio); CLL B cell viability was assessed at 3, 5 and 7 days using Annexin V staining. As shown in Figure 1A, leukemic B cells underwent apoptosis when cultured in medium alone; their survival was rescued following co-culture with MSCs (13.3%±13.2% CLL B cells in medium alone *vs* 59.2%±17.1% in presence of MSCs, after 7 days; *p* < 0.0001). The same pro-survival effect was observed using both autologous and allogeneic MSCs (data not shown). In addition, MSCs displayed a higher protective effect with respect to the human stromal cell line HS-5 (59.2%±17.1% with MSCs *vs* 45.9% ± 15.4% with HS-5; *p* < 0.01). After 7 days of culture, normal B cell survival with MSCs was 34.9%±15.7% *vs* 6.7%±4.3% in medium alone (*p* < 0.001) (Figure 1B), pointing out that MSC display a major protective effect on malignant B cells. MSCs from healthy donors rescued CLL B cells from apoptosis as well as MSCs from CLL patients (data not shown).

**Table 1:** Clinical characteristics of CLL patients studied for MSCs isolation

MSCs #	RAI stage	Age	Sex	Bone marrow infiltration (% Lymphocytes)	CD19/5 (%)
#01	0	75	M	34	71
#02	1	65	M	70	92
#03	1	78	M	87	92
#04	0	66	F	70	83
#05	1	70	M	90	98
#06	3	75	M	40	68
#07	0	50	F	18	1
#08	0	49	M	56	72
#09	2	60	F	5	2
#10	2	72	M	75	46
#11	1	68	M	13	11
#12	2	61	M	26	54
#13	2	55	M	6	8
#14	0	51	M	54	86
#15	2	47	M	78	86
#16	2	59	F	19	34
#17	0	66	M	40	75
#18	0	54	M	42	68
#19	0	51	M	27	40
#20	1	48	M	47	83
#21	1	55	M	52	70
#22	1	55	M	18	60
#23	0	49	M	62	81
#24	1	69	M	56	50
#25	0	51	M	37	54
#26	1	66	M	64	70
#27	1	60	F	43	56
#28	0	60	M	37	54
#29	1	70	F	70	50
#30	1	63	M	43	70
#31	0	63	M	55	67
#32	1	60	M	40	43
#33	0	56	F	52	45
#34	0	71	F	25	22
#35	0	49	F	62	69
#36	0	60	M	75	85
#37	0	58	F	60	43
#38	3	59	F	50	77
#39	2	72	F	75	85
#40	2	55	M	17	35
#41	2	63	M	90	93
#42	0	56	F	32	50
#43	1	57	M	34	91
#44	0	52	M	21	44
#45	2	67	F	73	81
#46	1	56	F	40	48

### CLL clones show different sensitivity to MSC pro-survival signals

**Figure 2:**
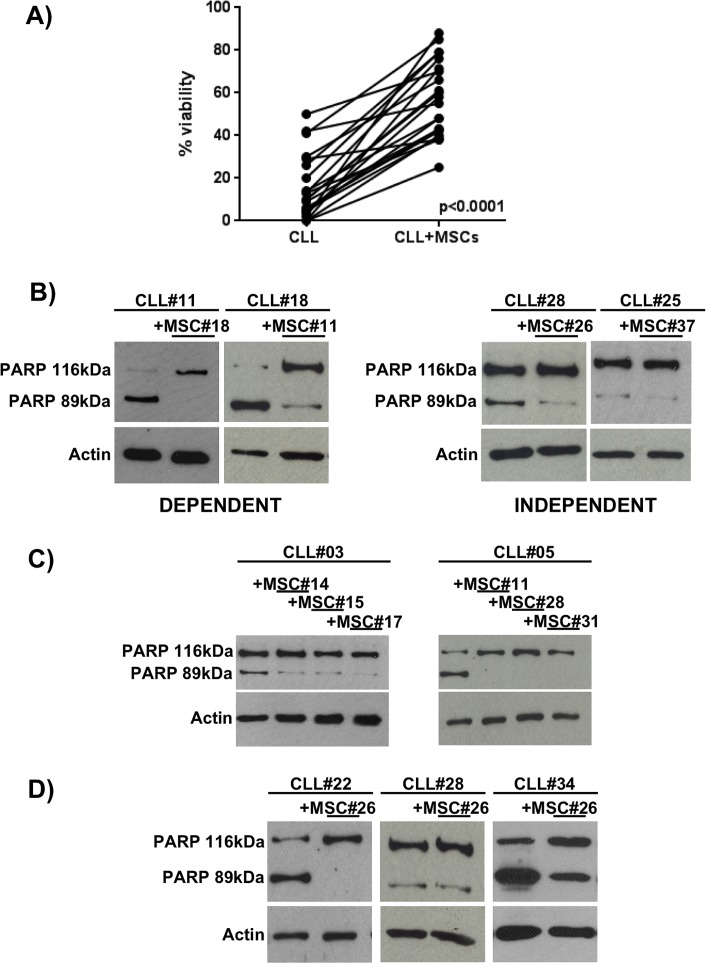
CLL B cell viability after 7 days of co-culture with MSCs **A.** Leukemic B cell viability, assessed by flow cytometry using Annexin V staining, demonstrated a high heterogeneity in response to MSCs pro-survival stimuli. Student's paired *t* test; *p* < 0.0001. **B.** The total cell lysates were subjected to SDS-PAGE, transferred to nitrocellulose membrane and detected with anti-PARP and anti-β-actin. The figure shows a representative case of neoplastic B cells obtained from CLL patients (CLL#) co-cultured with MSCs (MSC#); CLL clones displaying the cleaved PARP after 7 days of culture, but showing the full length protein in presence of MSCs were classified as dependent (*n* = 15, left panel). CLL clones displaying the full length PARP with and without the presence of MSCs were classified as independent (*n* = 12, right panel). **C.** Representative cases of CLL B cells (CLL#) co-cultured with MSCs derived from different CLL patients (MSC#), demonstrating that neoplastic B cells present the same PARP pattern with different MSC lines. **D.** CLL B cells from distinct patients exposed to the effect of the same MSC line showed different degree of dependency to MSC pro-survival stimuli.

Considering the heterogeneity of CLL B cell viability in the presence of MSCs (Figure 2A), we evaluated the PARP cleavage (generating an 89kDa fragment) as a marker of apoptosis in 27 CLL patient samples after 7 day co-culture of leukemic B cells with and without MSCs. On the basis of PARP cleavage we identified two different patterns: 1) one observed in a group of 15 patients in which leukemic B cells died following culture in medium alone, while they were rescued from apoptosis following culture with MSCs (the group was defined as “dependent” from microenvironment, Figure 2B, left panel); 2) a second pattern observed in a group of 12 CLL patients in which the addition of MSCs did not affect leukemic cell viability (the group defined “independent” from microenvironment, Figure 2B, right panel). We showed that this behaviour is not due to MSC features, since we did not find any differences using MSCs obtained from different patients (Figure 2C), while three different CLL clones exhibited different pattern of PARP cleavage in the presence of the same MSC line (completely abrogated in the first and second clone and still present in the third, Figure 2D). Finally, we correlated the 15 dependent and 12 independent patients with some prognostic factors for CLL, such as IgV_H_ mutational status, CD38 and ZAP70 expression, and genomic aberrations (13q-, 17p-, 11q- deletion and 12+) (Figure S2). We did not observed any significant correlation between the different groups of patients. Clinical and biological features of patients are reported in Table 2.

**Table 2:** Clinical characteristics of CLL patients

CLL#	Lymphocytes doubling time	RAI stage	Cytogenetic^1^	IgVH mutational status (M/UM)^2^	ZAP70^3^	CD38^4^
#01	>6months	0	na	M	pos	neg
#02	>6months	1	na	M	neg	neh
#03	>6months	2	13q-	M	nd	nd
#04	>6months	4	13q-	UM	pos	pos
#05	>6months	1	na	M	nd	pos
#06	>6months	1	11q- 12+	UM	pos	nd
#07	>6months	1	13q-	UM	neg	pos
#08	>6months	2	na	M	nd	nd
#09	>6months	2	17p- 13q-	nd	nd	nd
#10	>6months	0	13q-	M	neg	neg
#11	>6months	0	N	UM	pos	neg
#12	>6months	0	13q-	UM	neg	neg
#13	stable	1	13q-	M	pos	neg
#14	>6months	2	na	UM	pos	pos
#15	stable	0	13q-	M	pos	neg
#16	<6months	1	12+	UM	pos	pos
#17	<6months	1	17p-	UM	neg	nd
#18	>6months	4	17p-	UM	neg	pos
#19	stable	1	13q-	M	nd	nd
#20	stable	0	17p- 13q-	M	neg	neg
#21	>6months	1	13q-	M	0	neg
#22	stable	1	11q- 13q- 12+	UM	pos	pos
#23	na	1	na	M	na	na
#24	>6months	1	11q- 12q-	UM	pos	neg
#25	<6months	0	N	UM	pos	neg
#26	>6months	1	N	M	pos	neg
#27	stable	0	13q-	UM	pos	neg
#28	stable	1	13q-	M	neg	pos
#29	na	2	13q-	UM	pos	pos
#30	na	0	13q-	M	pos	pos
#31	stable	4	na	M	pos	neg
#32	<6months	4	11q-	UM	pos	neg
#33	stable	0	N	UM	pos	neg
#34	stable	0	N	M	pos	nd
#35	stable	1	13q-	M	pos	neg
#36	stable	0	11q- 13q- 12+	UM	pos	pos
#37	<6months	1	11q- 12+	nd	pos	pos
#38	>12months	0	nd	UM	nd	nd
#39	stable	1	13q-	M	pos	neg
#40	<6months	2	11q- 13q-	UM	neg	pos
#41	stable	1	13q-	M	neg	neg
#42	na	nd	13q-	M	neg	neg
#43	nd	nd	12+	na	neg	neg
#44	>6months	2	11q-	UM	neg	pos
#45	na	nd	na	na	na	pos

1 N = normal karyotype.

2 M=mutated, defined as having a frequency of mutations greater than 2% from germline VH sequence.

3 As determined by cytofluorymetric analysis (cut-off: 20%).

4 As determined by cytofluorymetric analysis (cut-off: 30%).

na= not available, nd=not determined

### MSCs affect CLL B cell behavior through the release of soluble factors and cell-cell contact

**Figure 3:**
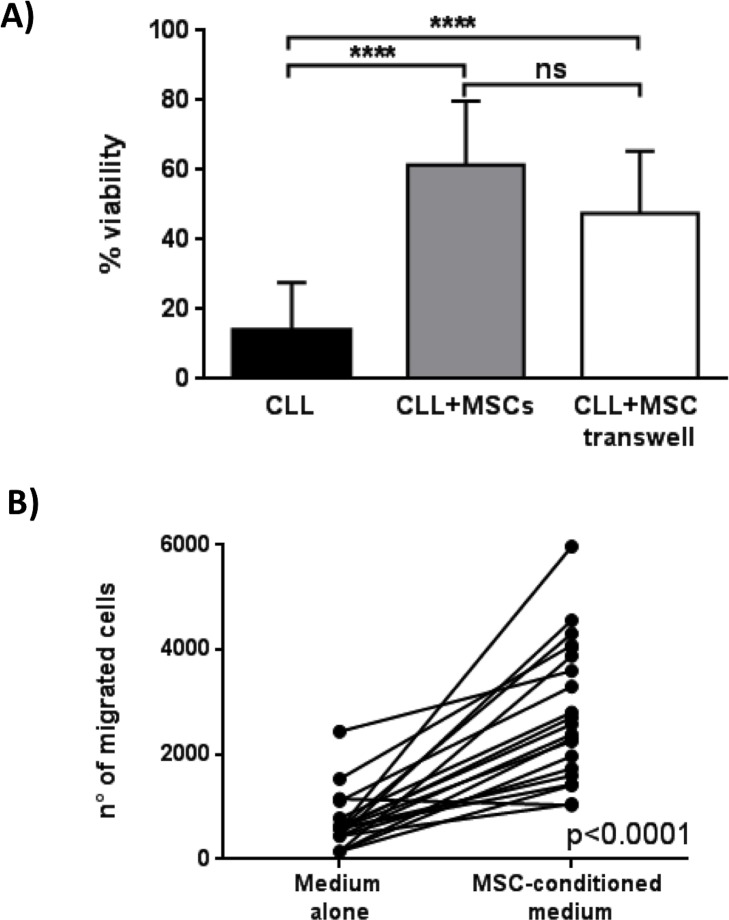
Effect of MSCs on CLL B cell viability and migration **A.** Viability of leukemic B cells cultured alone (CLL), with MSCs in direct contact (CLL+MSCs) and in presence of a 0.4μm transwell system (CLL+MSC transwell) was assessed after 7 days by flow cytometry using Annexin V staining. Data are presented as mean ± standard deviation for 12 separated experiments; paired Student's *t* test; ****p < 0.0001. **B.** CLL B cells (*n* = 21) were allowed to migrate through a 3μm porous filter towards medium alone or MSC-conditioned medium for 3h; paired Student's *t* test; ****p < 0.0001.

To discriminate between the role of cell-cell contact and soluble factors in the anti-apoptotic effect of MSCs, we cultured CLL B cells in direct contact to MSCs or in presence of MSCs separated by a porous transwell (TW) insert. After 7 days of culture, the viability of 12 CLL samples co-cultured with MSCs both in direct contact and following TW was 61.5%±18.3% and 47.5%±7.8% (*p* = 0.07), respectively, suggesting that both cell-contact and soluble factors contribute to the anti-apoptotic effect (Figure 3A). As cell migration is concerned, we demonstrated that the presence of MSC-conditioned medium (MSC-CM) significantly increases CLL B cell migration (651±543 migrated cells in medium alone *vs* 2,809±1,318 migrated cells in MSC-CM, *p* < 0.0001) (Figure 3B).

### CLL B cells modulate the cytokine secretion profile of MSCs

**Figure 4:**
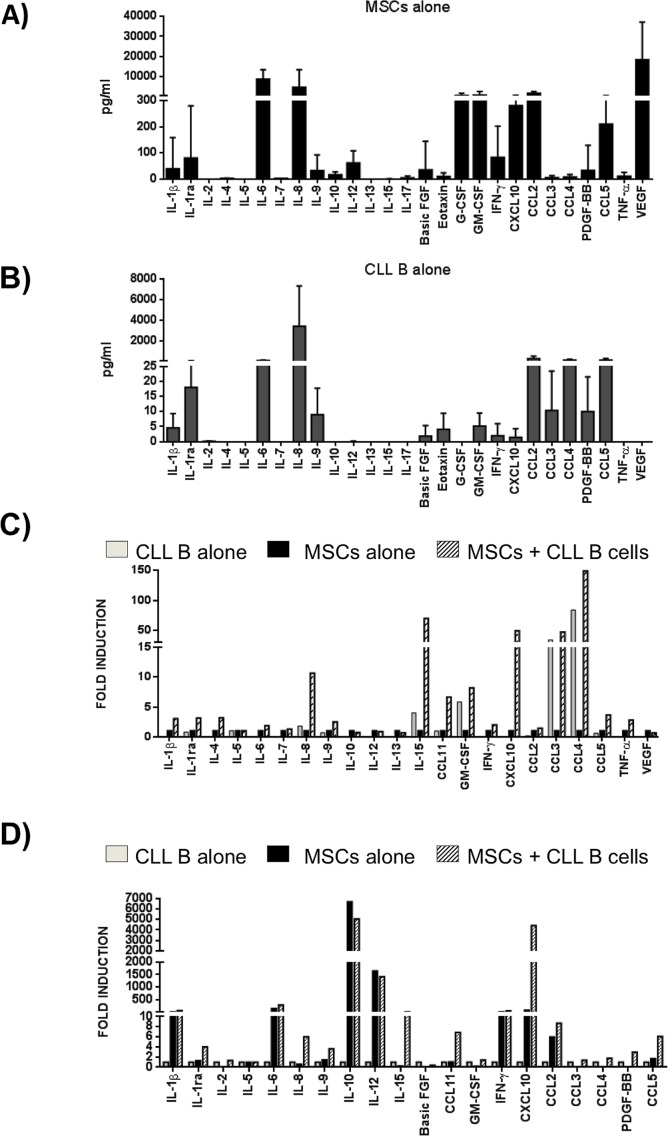
Cytokine/Chemokine secretion profile **A.** MSCs and **B.** CLL B cells were cultured in medium alone for 7 days and the level of cytokines in supernatants was assessed by Bio-Plex (*n* = 11). Data are expressed as mean ± standard deviation of the concentration in pg/ml. **C.-D.** MSCs and CLL B cells were cultured in direct contact in complete medium for 7 days (*n* = 4). Cytokines/chemokines quantification in supernatants was normalized both on the levels observed in the media of MSCs cultured alone **C.** and of CLL B cells cultured alone **D.**. The co-culture induced a general modulation for most of C/C tested. Data are expressed as Fold Induction, FI.

In order to better define the signals exchanged between leukemic B cells and MSCs, a multiplex cytokine analysis was performed on supernatants obtained following culture of CLL B cells alone, MSCs alone and a combination of CLL B cell with MSCs. The quantification of the secretoma of MSCs and neoplastic B cells alone is reported in Figure 4A and 4B, respectively. The CLL B cell/MSC co-cultures induce an increase (expressed as Fold Induction, FI) for most of the assessed molecules, being more evident for IL-8, CXCL10, CCL4 and CCL11. Particularly, these four molecules resulted up-regulated normalizing their concentration both on supernatant of MSCs alone (IL-8 10.6 FI, CCL4 148.6 FI, CCL11 6.7 FI and CXCL10 49.7 FI, Figure 4C) and on supernatant of CLL B cells alone (IL-8 5.8 FI, CCL4 1.8 FI, CCL11 6.9 FI and CXCL10 4411 FI, Figure 4D).

**Figure 5:**
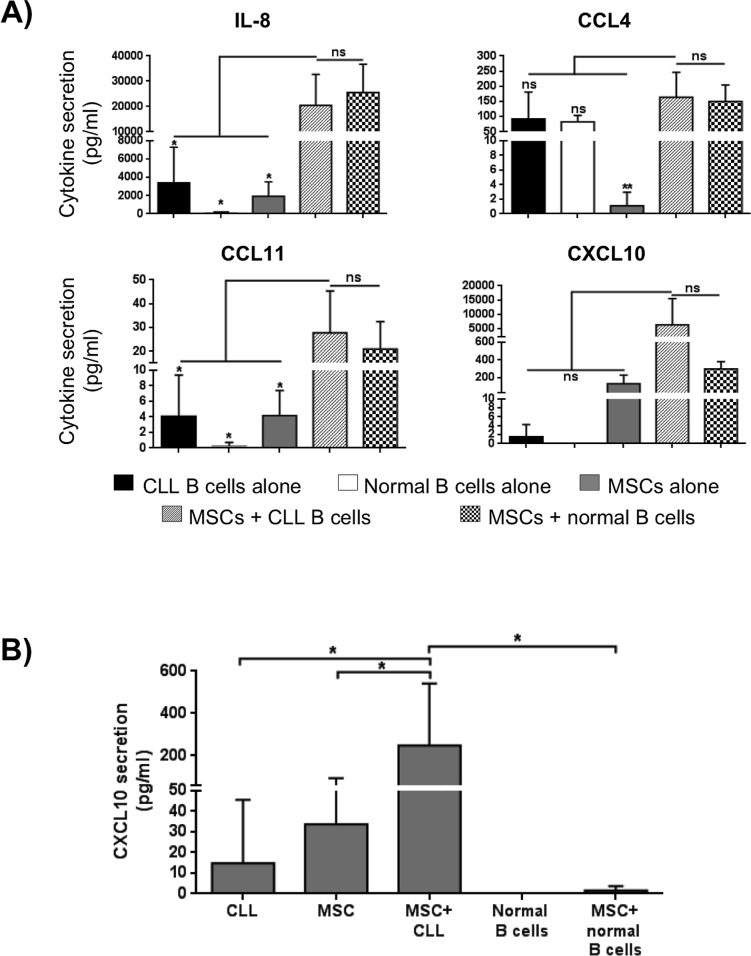
Modulation of *in vitro* soluble factor production under different culture conditions **A.** Quantification in supernatants was assessed by Bio-Plex; under co-culture conditions, IL-8, CCL4 and CCL11 reached a concentration higher than concentration detected in supernatant of the three single cell-population examined (MSCs, CLL and normal B cells; *p < 0.05, **p < 0.01), but similar to the concentration detected co-culturing normal B cells in the presence of MSCs. On the contrary, CXCL10 production resulted high in supernatant of MSCs co-cultured with neoplastic B cells, but not with B cells from healthy subjects. **B.** MSCs were cultured in direct contact with CLL B cells in complete medium for 7 days (*n* = 7). CXCL10 quantification in supernatants was determined by ELISA assay. ANOVA test; *p < 0.05.

Interestingly, the above mentioned cytokines reached similar concentrations when the co-cultures were carried out in the presence of leukemic and normal B cells (Figure 5A). The only exception is represented by CXCL10, whose up-regulation was detected only during MSC/CLL B cell co-cultures, as confirmed by ELISA assay (Figure 5B). Finally, we tested the modulation of the secretion profile of leukemic B cells culture in the presence of MSC conditioned medium (MSC-CM) to assess how the factors released by MSCs can influence CLL B cell cito/chemokine production. We found that MSC supernatant induced an increase of different molecules, particularly IL-8 (27.4 FI), CCL4 (149 FI), CXCL10 (40 FI) and CCL5 (9.9) (Figure S3).

### MSCs from CLL patients protect leukemic B cells after *in vivo* treatment with fludarabine and cyclophosphamide containing regimen

**Figure 6:**
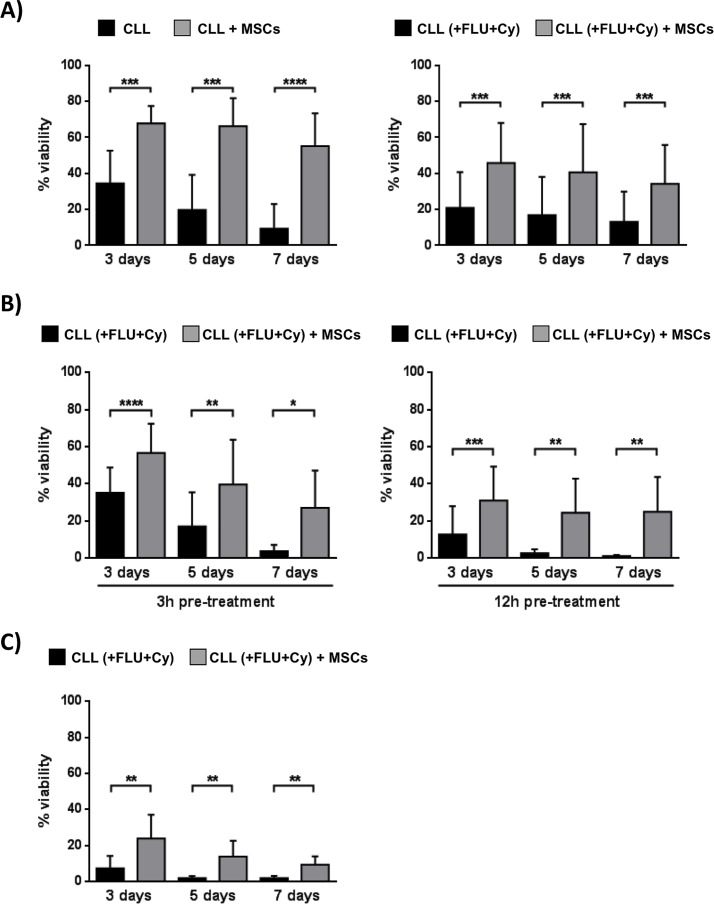
MSCs protect CLL B cells from *in vivo* drug-induced apoptosis **A.** CLL B cells were collected before and at the 3^rd^ day of the first *in vivo* treatment with FLU and Cy, according to the FLU-Cy protocol, and cultured in presence of MSCs (*n* = 10). Viability was measured at the time point of 3, 5 and 7 days by Annexin V staining. MSCs provided a significant level of protection from apoptosis in CLL B cells both pre- (left panel) and post-treatment with FLU-Cy (right panel) when compared to neoplastic B lymphocytes in medium alone. **B.** CLL B cells purified from 8 untreated patients were pre-treated for 3 hours (left panel) and 12 hours (right panel) with FLU-Cy or directly without pre-treatment **C.** and then cultured in the presence or absence of MSCs for 3, 5 and 7 days. MSCs preserved CLL B cells from *in vitro* drug-induced apoptosis. Data are presented as mean ± standard deviation; paired Student's *t* test; *p < 0.05, **p < 0.01, ***p < 0.001, ****p < 0.0001.

MSCs protect leukemic B cells from apoptosis induced *in vitro* by Fludarabine, Cyclophosphamide, Bendamustine, Prednisone and Hydrocortisone [28]. We used an *in vitro* co-culture system that employed leukemic B cells obtained from 10 CLL patients before and after one cycle of Fludarabine (FLU)/Cyclophosphamide (Cy) therapy. As shown in Figure 6A, the presence of MSCs provide a significant protection from apoptosis in CLL B cells with respect to neoplastic cells cultured in medium alone; the viability ranged from 55.2%±18.2% with MSCs *vs* 9.4%±13.6% without MSCs, *p* < 0.0001, before *in vivo* treatment and from 34.2%±21.6% with MSCs *vs* 13.1%±16.7% without MSCs, *p* < 0.0001, after *in vivo* therapy.

At the same time, we performed an *in vitro* parallel experiment assessing the MSC protective role on CLL cells exposed to the same compounds used for *in vivo* therapy. Leukemic B cells purified from 8 CLL untreated patients were pre-incubated for 3 and 12 hours with FLU and Cy and then cultured in the presence or absence of MSCs for 3, 5 and 7 days. Also in this case (Figure 6B), MSCs preserved CLL B cells from drug-induced apoptosis (after 3h pre-treatment and 7 day co-culture, neoplastic B cell viability, 27%±20% with MSCs *vs* 3.6%±3.4% without MSCs, *p* < 0.05; after 12h pre-treatment and 7 day co-culture, 25.1%±20.3% with MSCs *vs* 0.86%±0.69% without MSCs, *p* < 0.05). The same pattern was observed following co-culture of CLL B cells and MSCs with FLU+Cy for 3, 5 and 7 days without pre-treatment (after 7 days, neoplastic B cell viability, 9.8%±4.5% with MSCs *vs* 1.8%±1.3% without MSCs, *p* < 0.01) (Figure 6C).

### MSCs from CLL patients do not protect leukemic B cells from apoptosis induced by kinase inhibitors

**Figure 7:**
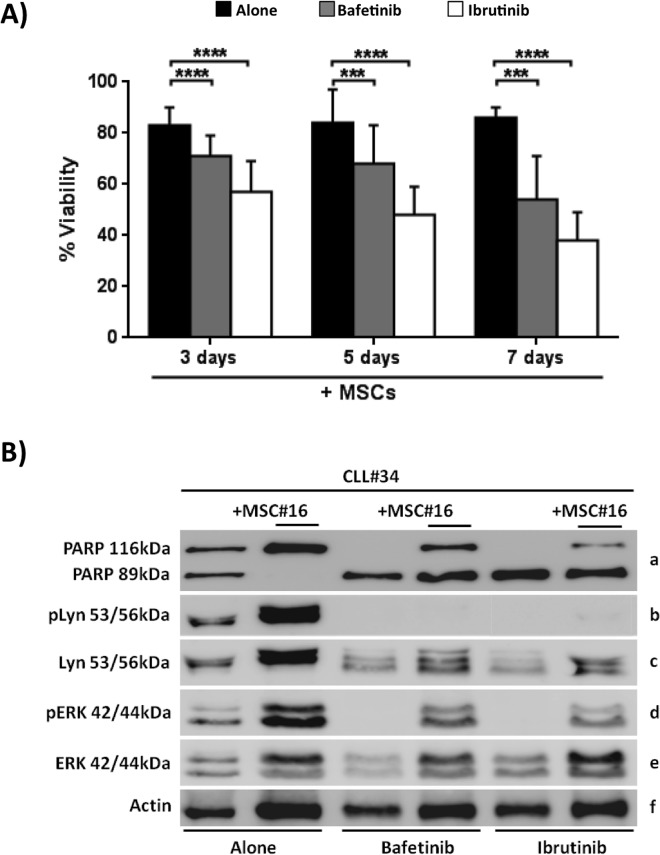
Kinase inhibitors counteract the MSC pro-survival effect CLL B cells were cultured with MSCs in presence of Bafetinib and Ibrutinib. **A.** CLL B cells were co-cultured for 3, 5 and 7 days at the following conditions: medium alone, with the addition of 5μM Bafetinib and 5μM Ibrutinib. The graphs show the mean ± standard deviation of Annexin V^neg^ cells percentage of 12 separated experiments compared with untreated controls; paired Student's *t* test; ***p < 0.001; ****p < 0.0001. **B.** The total cell lysates (*n* = 10) were subjected to SDS-PAGE, transferred to nitrocellulose membrane and detected sequentially with: anti-PARP (row a), anti-Lyn-Tyr396(row b), anti-Lyn (row c), anti-ERK-Thr202/Tyr204 (row d), anti-ERK (row e) and anti-β-actin (row f). The figure shows a representative case of CLL B cells (CLL#) treated with 5μM Bafetinib and 5μM Ibrutinib and cultured with and without MSCs (MSC#).

Previous studies [28, 29] and our data above reported demonstrated that MSCs can protect CLL B cells from drug-induced apoptosis. We next studied the effect of Bafetinib and Ibrutinib on the survival of neoplastic B cells obtained from 12 CLL patients cultured with and without MSCs. Leukemic B cell viability was assessed at 3, 5 and 7 days using Annexin V staining. We found that the presence of MSCs was not enough to inhibit Bafetinib and Ibrutinib induced apoptosis of neoplastic B cells (Figure 7A). In fact, after 7 day co-cultures, CLL B cell viability was 85.7%±4.1% in the absence of kinase inhibitors, 53.9%±17.2% in the presence of Bafetinib (*p* < 0.001), and 37.7%±14.7% with Ibrutinib (*p* < 0.0001). The data were also confirmed by western blotting analysis of PARP protein, indicating that, although the presence of MSCs, the addition of Bafetinib and Ibrutinib induced leukemic B cell *in vitro* apoptosis (Figure 7B, row a). To better understand the molecular mechanism by which the two inhibitors affect neoplastic B cell survival, we evaluated the phosphorylation status of Lyn (the tyrosine kinase that sustains the neoplastic clone survival through its constitutive activation) [30, 31] and ERK (a protein involved in BCR signaling, CXCL12/CXCR4 axis and responsible for the reduced apoptosis and the migration of CLL cells beneath stromal cells) [32]. We found that the treatment with Bafetinib considerably turns off Lyn phosphorylation at Tyr396 active site although the presence of MSCs; the same effect was also observed after treating CLL B cells with Ibrutinib (Figure 7B, row b). Both inhibitors are able to reduce ERK phosphorylation in leukemic B cells cultured with MSCs with respect to untreated controls (Figure 7B, row d).

Moreover, we determined the effect of both kinase inhibitors on the survival of CLL B cells following engagement of BCR with anti-IgM. The pre-incubation of CLL B cells with Bafetinib and Ibrutinib reduced cell viability (Figure S4A); the latter data were confirmed by western blotting analyses of PARP, Lyn and ERK (Figure S4B).

In addition, on the base of the cleavage pattern of PARP protein, we divided the 12 patients treated with Bafetinib and Ibrutinib into “dependent” and “independent” groups showing no differences between the two groups on CLL B cell viability and Lyn as well as ERK de-phosphorylation (data not shown). This set of experiments suggest that kinase inhibitors mediated cytotoxicity independently from the protective effect of the microenvironment.

### Bafetinib and Ibrutinib do not reduce neoplastic B cell migration to bone marrow stroma

**Figure 8:**
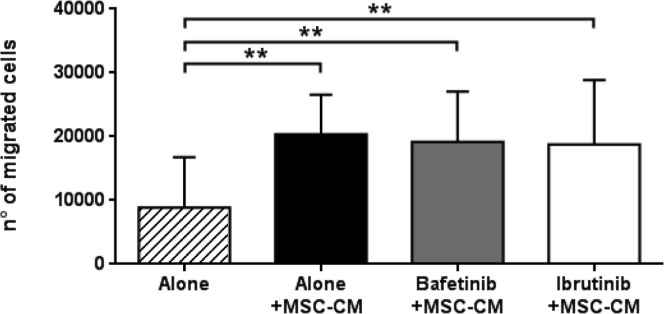
MSC-conditioned medium preserves CLL B cell migration despite kinase inhibitors treatment CLL B cells (*n* = 10) were pre-treated with 5μM Bafetinib and 5μM Ibrutinib for 1h and then allowed to migrate through a 3μm porous filter towards medium alone or MSC-conditioned medium for 3h; paired Student's *t* test; **p < 0.01with respect to untreated cells.

By chemotaxis assay, we evaluated the effect of Bafetinib and Ibrutinib kinase inhibitors on the cross-talking between CLL B cells and bone marrow MSCs. The pre-treatment of neoplastic B cells with Bafetinib and Ibrutinib did not inhibit the chemotaxis induced by MSC-CM and the number of migrated cells significantly increased with respect to basal migration (8,858±7,920 alone *vs* 20,391±6,184 alone+MSC-CM, 19,517±7,931 Bafetinib+MSC-CM and 18,772±10,094 Ibrutinib+MSC-CM; *p* < 0.01, Figure 8).

Interactions between chemokines and their receptors mediate cell migration. CLL B cells express high levels of CXCR4, CCR7, CXCR5 and CXCR3 chemokine receptors [33, 34]. We investigated whether kinase inhibitors regulate chemokine receptor expression on neoplastic B cells cultured in the presence and absence of MSCs. The two kinase inhibitors affect receptor expression in different ways (Figure S5). In particular, CXCR4 was increased by the treatment with Bafetinib and Ibrutinib, while CCR7 was reduced by Bafetinib; at the same time, CXCR5 was affected only by Ibrutinib and the two kinase inhibitors do not show a significant influence on CXCR3 expression, suggesting that their action may be carried out mostly by the receptors involved in the process of CLL homing. The data are expressed as MFI and summarized in Table 3.

**Table 3:** Expression level of chemokine receptors on CLL B cells

	no MSCs			with MSCs		
	Alone (MFI)	Bafetinib (MFI)	Ibrutinib (MFI)	Alone (MFI)	Bafetinib (MFI)	Ibrutinib (MFI)
**CXCR4**	19,880±4,858	28,568±5,563 *p* < 0.01	24,442±6,105 ns	8,561±5,513	18,009±7,154 *p* < 0.0001	13,776±6,374 *p* < 0.0001
**CCR7**	5,085±1,308	3,475±988 *p* < 0.05	4,498±890 *p* < 0.05	5,229±1,237	3,845±609 *p* < 0.001	4,904±1,040 ns
**CXCR5**	1,697±590	1,712±600 ns	1,245±402 *p* < 0.01	1,623±507	1,621±466 ns	1,269±353 *p* < 0.05
**CXCR3**	3,969±2,635	2,379±1,721 ns	2,366±1,704 ns	4,405±2,601	3,053±2,374 ns	2,841±1,994 ns

### Bafetinib and Ibrutinib inhibit pseudoemperipolesis in CLL B cell/MSC co-cultures in a CD49d-dependent way

**Figure 9:**
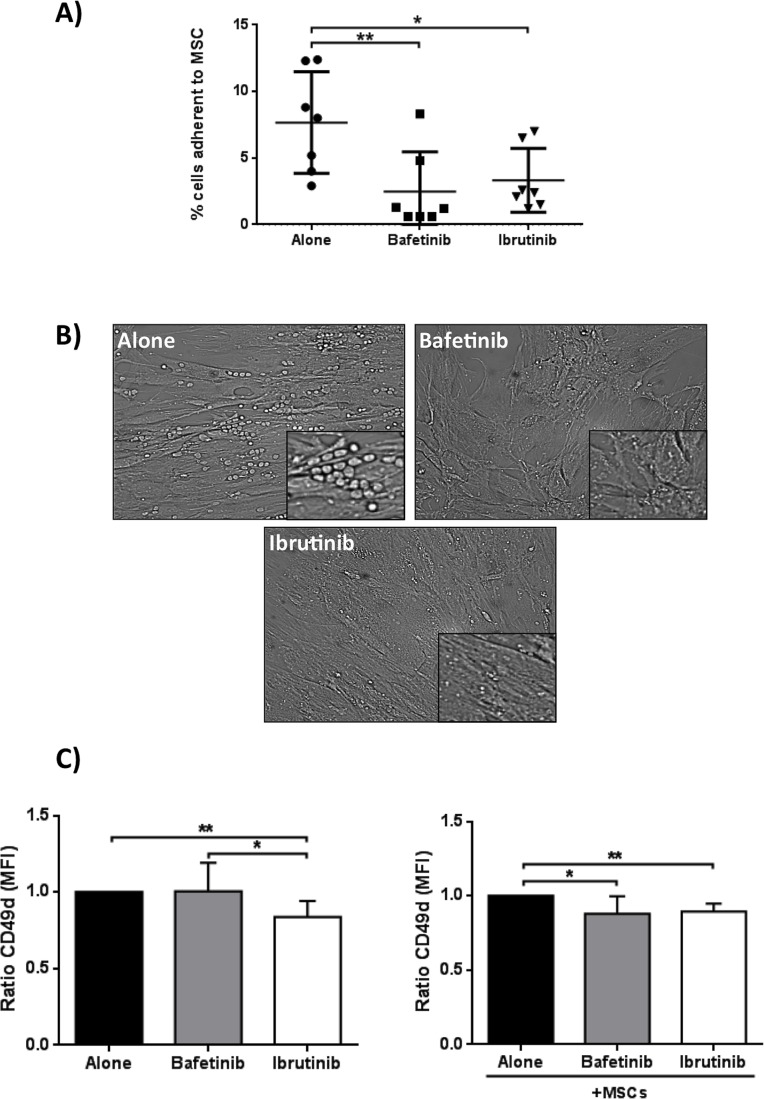
Reduction of spontaneous migration of CLL B cells beneath MSCs (pseudoemperipolesis) and CD49d surface expression **A.** CLL B cells were pre-treated for 1h with 5μM Bafetinib and Ibrutinib and seeded onto confluent marrow stromal cell layers. After overnight incubation, the CLL cells that did not migrated into the stromal cell layer were washed off. Graph represents the mean ± standard deviation of the percentage of migrated CLL cells from 7 different patients; paired Student's *t* test; *p < 0.05, **p < 0.01. **B.** Representative phase-contrast photomicrographs of pseudoemperipolesis of untreated CLL B cells (alone) and, in comparison, reduced pseudoemperipolesis of the same CLL sample pre-treated with Bafetinib and Ibrutinib (20X magnification, Olympus IX81). **C.** Malignant B cells (*n* = 7) were cultured alone or with MSCs for 24h and CD49d expression level was assessed by flow cytometry. Data are presented as the ratio of the mean ± standard deviation of MFI; paired Student's *t* test; **p < 0.01 with respect to untreated cells.

We evaluated the ability of neoplastic B cells to migrate and adhere beneath MSCs after treatment with Bafetinib and Ibrutinib. We found that the two inhibitors were able to significantly reduce CLL B cell pseudoemperipolesis. In fact, the percentage of neoplastic B cells adherent to the stromal layer was 7.7%±3.8% in the absence of inhibitors, 2.5%±3.0% with Bafetinib (*p* < 0.01), and 3.3%±2.4% with Ibrutinib (*p* < 0.05) (Figure 9A). The phase-contrast photomicrographs displayed in Figure 9B illustrate the effect of the pre-treatment of CLL B cells with the two molecules, highlighting a marked reduction of neoplastic cell adhesion to the MSC layer in the presence of inhibitors as compared to untreated controls (alone). Since CD49d plays a critical role in cell adhesion and increases the ability of malignant cells to access to protective niches [35], we addressed the effect of the two kinase inhibitors on its expression on CLL B cell surface, demonstrating that the treatment with Bafetinib and Ibrutinib significantly decreases CD49d expression in co-culture with MSCs (MFI ratio 0.87±0.12 Bafetinib, *p* < 0.05, and 0.89±0.05 Ibrutinib, *p* < 0.01) (Figure 9C).

## DISCUSSION

In this study MSCs from bone marrow of 46 CLL patients, co-cultured to mimic the neoplastic *in vivo* microenvironment, were isolated and characterized. We demonstrated that MSCs support *in vitro* leukemic B cell survival more efficiently than the stromal cell line HS-5 [36]. In the last years, several studies tried to elucidate the role of stromal cells in CLL B cell survival, and primary bone marrow stromal cells have been shown to be effective in inhibiting apoptosis of cultured CLL [37] and acute lymphocytic leukemia (B-ALL) cells [38]. In these reports, the *in vitro* co-culture systems used were represented by layers of marrow cells, including fibroblasts, macrophages and fat cells, or layers of immortalized cell lines, but not from a selected and well defined cell population.

Our data show that CLL B cells are susceptible to the anti-apoptotic effect of MSCs, favoring neoplastic B cell survival *in vitro* for at least 7 days. These results are noteworthy considering that CLL B cells spontaneously undergo apoptosis once they have been removed from the *in vivo* microenvironment and placed in suspension cultures without supportive stroma. Despite CLL B cells display typical phenotype and cytogenetic abnormalities, the disease is quite heterogeneous in terms of clinical courses, suggesting that each leukemic clone is characterized by intrinsic features that might affect the interactions with the surrounding environment. In this context, we found a great variability in CLL cells survival when co-cultured with MSCs, since the fraction of living leukemic B cells ranged from 40% to 76%. The analysis of the cleavage pattern of PARP protein detected in CLL patients after 7 day co-culture with MSCs allowed us to distinguish patients into two groups on the basis of their dependence/independence to microenvironmental pro-survival stimuli. The first group is represented by those CLL B cell samples that underwent spontaneous apoptosis in medium alone, but were rescued from apoptosis following exposure to MSCs. On the contrary, the second group identified CLL samples whose viability was high when cultured both in medium alone and in the presence of MSCs, indicating that these CLL clones were able to survive independently from signals coming from the microenvironment. In terms of survival, we observed that each CLL clone responded in the same manner to different MSCs samples, suggesting that the heterogeneity of leukemic clones is likely to be related to intrinsic features of leukemic B cells rather than to external stimuli.

The increase of leukemic B cell survival in the presence of MSCs might be mediated by soluble factors and/or cell-cell contact. Our results demonstrated that both soluble factors and cell-cell contacts are able to support neoplastic B cell viability. Survival factors counteracting *in vitro* apoptosis of CLL B cells have been reported in the past [13, 39], but the humoral/cellular factors responsible for the pro-survival effect *in vivo* had not been yet identified. By a multiplex cytokine assay, we found that the MSC/leukemic cell co-culture system induced an increase for many of the cytokines/chemokines tested, the strongest being represented by IL-8, CCL4, CCL11 and CXCL10. Although the involvement of IL-8 and CCL4 in CLL leukemic cell survival and growth has been already reported in the literature [40-44], our findings confirm these data and extend the observations on CCL11 and CXCL10 production. In particular, the addition of MSCs to leukemic B cells up-regulates the level of these two molecules. Regarding CCL11, this effect is not a peculiar finding in malignant B cells since normal B cells showed the same pattern of secretion; more interestingly, CXCL10 is exclusively produced when MSCs were co-cultured with malignant B cells but not with normal B lymphocytes, supporting a key role for this chemokine in CLL progression. The basal production of CXCL10, in fact, was very low in the supernatants of CLL leukemic B cells and of MSCs cultured alone, while we found a marked increase after co-culture. In light of the fact that CXCL10 receptor, CXCR3, is constitutively expressed on CLL B cell surface [33], these data stress the importance of the interconnection between chemokine receptor signaling and stimuli coming from the microenvironment.

Furthermore, we demonstrated that MSCs were able to rescue leukemic B cells from apoptosis also when they have been previously exposed to *in vivo* Fludarabine (FLU) and Cyclophosphamide (Cy) containing regimen. These data point out the protective role of microenvironment, not only toward neoplastic *in vitro* B cells [28], but also on apoptosis induced by the *in vivo* administration of the chemotherapy.

In the last few years, the observation of the key role of tumor microenvironment and immune dysfunctions in leukemic cells accumulation prompted the use of a number of compounds targeting CLL microenvironment. Different drugs used for CLL treatment have been demonstrated to induce *in vitro* apoptosis of CLL B cells whereas their effect is reduced when administered *in vivo*, probably related to the presence of pro-survival stimuli coming from surrounding environment. Therefore, it is mandatory to test the effect of new therapeutic agents in the presence of microenvironmental partners (such as MSCs) which might interfere with the biological effects of the drug, allowing the identification of subgroups of patients who may benefit from treatments targeting the cross-talk with supportive cells at the sites of the disease. For this reason, we tested in our co-culture system the effect of inhibitors targeting different kinases involved in BCR pathway, such as Bafetinib and Ibrutinib, the latter known to reduce CLL B cell migration and to induce *in vitro* apoptosis [23, 45]. We found that both Bafetinib and Ibrutinib were able to induce leukemic cell apoptosis independently from MSCs presence, through a mechanism involving a de-phosphorylation of Lyn and ERK kinases, two relevant proteins in CLL pathogenesis. In contrast, these inhibitors did not affect B cell migration toward MSC-conditioned medium, rich in cytokines and chemokines, suggesting that these cells do not lose their ability to move toward a protective niche. Migration is mediated by the interaction between chemokines and their receptors, i.e. CXCR4, CCR7, CXCR5 and CXCR3, these latter being overexpressed on CLL B cells [33, 34]. In this work, we observed that, in malignant B cells co-cultured with MSCs, Bafetinib significantly decreased CCR7 expression, involved in lymphocyte entry the lymph node, and up-regulated CXCR4 which, instead, is engaged in the trafficking in lymphoid organs. CXCR4 up-regulation was also observed following *in vitro* treatment with Ibrutinib. Although it still remains unknown the exact mechanism of CXCR4 up-regulation, we found that the use of kinase inhibitors induced modifications in chemokine receptors. Since cell-cell contact with stromal cells is crucial for CLL B cell survival, we tested the effect of Bafetinib and Ibrutinib on neoplastic B cell adhesion to an MSC layer finding a significant reduction of cell adhesion through a down-modulation of CD49d expression. Taking together, these results suggest that Bafetinib and Ibrutinib not only are able to reduce CLL B cell viability by blocking molecules important for cell survival, but they could interfere with the interactions between chemokines and their receptors and with cell-cell contact.

In conclusion, this study demonstrates that MSCs co-culture represents a reproducible *in vitro* system with functional similarities to *in vivo* bone marrow conditions, pointing out that the heterogeneity of the disease is reflected also in CLL B cell capacity to respond to favorable signals from MSCs. Moreover, as a result of the ability of kinase inhibitors to inactivate enzymes in the BCR signaling pathway, which are aberrantly activated in CLL, leukemic B cells lose not only their ability to proliferate and survive but also to interact with the protective bone marrow microenvironment, a necessary condition to aim the eradication of the disease. In addition, for the first time, we herein demonstrated that Bafetinib presents a mechanism of action quite similar to that of Ibrutinib in blocking CLL B cell-MSC cross-talk. Our findings on the role of MSCs and their effect on neoplastic B lymphocytes open a new scenario to better identify the most effective drugs or drug combinations targeting the pro-survival cross-talk between neoplastic B cells and marrow elements.

## MATERIALS AND METHODS

### Ethic statement

Primary CLL samples were obtained after informed consent according to the Declaration of Helsinki. The ethic approval for our study was obtained from the local ethic committee of “Regione Veneto on chronic lymphocytic leukemia”.

### Mesenchymal stromal cell long-term cultures

Mesenchymal stromal cells (MSCs) were isolated from iliac crest bone marrow (BM) aspirate of 46 CLL patients (Table 1) under local anesthesia and diluted 1:3 in Phosphate Buffered Saline (PBS1X) (Euroclone; Milan, Italy). BM mononuclear cells (BMMCs) were isolated by F/H (Amersham Biosciences) centrifugation and plated at density 1,000cells/cm^2^ in Dulbecco's modified Eagle's medium (DMEM) (Euroclone) with 1,000mg/ml glucose, L-glutamine, 10% heated inactivated fetal bovine serum (FBS) and 100U/ml Penicillin, 100μg/ml Streptomicin (Life Technologies; Paisley, UK). BMMC suspensions were incubated at 37°C in humidified atmosphere containing 5% CO_2_ and allowed to attach for 7 days; at this time-point, non-adherent fraction was discarded and adherent cells were fed every week with fresh medium. These cells were maintained until confluence, then they were removed by Accutase (Sigma-Aldrich; Milan, Italy), centrifuged and diluted 1:3 for subsequent expansion in 25cm^3^ flasks or cryopreserved for future uses.

### Human primary samples

Peripheral blood mononuclear cells (PBMCs) were obtained from heparinised venous blood of 45 CLL patients (Table 2) and 11 normal donors, representative of the adult healthy population. PBMCs of the patients were isolated by density-gradient centrifugation over Ficoll-Hypaque (F/H; Amersham Biosciences; Buckinghamshire, UK). Where necessary, further purifications were performed. Finally all the samples utilized had a content of neoplastic B cells greater than 95%. Untouched peripheral blood B cells from normal donors were isolated using the RosetteSep isolation kit for B cells (STEMCELL Technologies; Vancouver, Canada). The purity of the obtained peripheral blood cells was at least 95% (CD19+), as assessed by flow cytometry. All patients had a confirmed diagnosis of CLL by NCI Working Group Definition [46] and were classified according to the Rai staging system [47].

### Culture conditions

Purified leukemic and normal B cells (2×10^6^/ml) and MSCs (1×10^5^/well seeded into 12 well plates) were cultured in complete RPMI-1640 medium (Sigma-Aldrich) at 37°C in a humidified atmosphere containing 5% CO_2_. For co-culture experiments, neoplastic and normal B cells were added to MSC layer at 20:1 ratio in complete RPMI-1640 medium. The plates were incubated at 37°C, in a humidified atmosphere containing 5% CO_2_ and B lymphocytes were harvested after 3, 5 and 7 days. In some experiments, B cells obtained from 10 CLL patients before and after therapy according to the protocol FLU-Cy: FLU 25 mg/m^2^ for 3 days and Cy 350 mg/m^2^ for 3 days, every 28 days; this therapy is the standard of care for patients needing a therapy. For *in vitro* treatment with FLU and Cy, CLL B cells were incubated with 20μM FLU and 5mM Cy, as previously described [48]. For experiments using kinase inhibitors, CLL B cells were cultured in presence and absence of MSCs, as previously described, adding 5μM Bafetinib (Selleck Chemicals; Munich, Germany), 5μM Ibrutinib (Selleck Chemicals), and/or goat F(ab')2 anti-human IgM (10μg/ml) (Southern Biotech; Birmingham, USA). The concentration of inhibitors is chosen according to preliminary experiments with different doses. In some experiments, we also employed the HS-5 cell line, a human fibroblastoid cell line available from the American Type Culture Collection (ATCC; Manassas, VA), which represent a component of the marrow microenvironment [49]. To achieve complete confluence and the formation of a stable feeder monolayer, 1×10^5^ HS-5 cells were plated in 12 well plates and irradiated at 4,000 Rad × 15 minutes at least 48-72 hours before the addition of leukemic B cells. Transwell (TW) experiments, using polycarbonate membranes (Corning Costar; Cambridge, UK) with 0.4μm pores, were performed to prevent physical contact between B lymphocytes and MSCs, allowing the diffusion of soluble factors secreted by the adherent layers of stromal cells.

### Cell viability testing

Freshly isolated CLL B and normal B cells were cultured in direct or indirect (TW) contact with MSCs and HS-5 at a ratio of 20:1. MSCs and HS-5 were cultured in 12 well plate with DMEM until confluence and then placed in RPMI medium (Euroclone), prior the addiction of B lymphocytes. Leukemic cells were collected after 3, 5 and 7 days, leaving intact the adherent layer, and examined for apoptosis status by staining with Annexin V-FITC accordingly to the manufacturer's instructions (Immunostep; Salamanca, Spain). Briefly, aliquots of 5×10^5^ cells were harvested, washed and incubated for 10 minutes in the dark and at RT with anti-CD19 APC (Invitrogen). Then, cells were washed and 100μl of binding buffer plus 5μl of Annexin V-FITC were added for further 10 minutes in the dark and at RT. After the incubation, 100μl of binding buffer were added and cells were analyzed by flow cytometer FACSCalibur. At least 20,000 events were collected using CellQuestPRO software.

### Multiplex cytokine analysis

In order to quantify chemokines and cytokines released in culture supernatants, cell-free culture media of previous experiments were collected after 7 days for the human Bio-Plex™ 27 plex Cytokine Assay (Bio-Rad Laboratories; Hercules, CA), which enables the detection of Interleukin-1β (IL-1 β), IL-1ra, IL-2, IL-4, IL-5, IL-6, IL-7, IL-8, IL-9, IL-10, IL-12 (p70), IL-13, IL-15, IL-17, Basic FGF, CCL11, G-CSF, GM-CSF, IFN-γ, CXCL10 (IP-10), CCL2 (MCP-1), CCL3 (MIP-1α), CCL4 (MIP-1β), PDGF-BB, CCL5, TNF-α and VEGF. Measurements were performed on Bio-Plex in duplicates. The Bio-Plex Manager™ software shows data as Median Fluorescence Intensity (MFI) as well as concentration (pg/ml). The concentration of the analyte bound to each bead is proportional to the MFI of reporter signals.

### ELISA assay

The cell-free culture media of previous experiments were collected after 7 days and analyzed for CXCL10 by ELISA (R&D Systems, Minneapolis, MN) according to the manufacturer's instructions.

### Western blotting

B cells (5×10^5^ for each assay) were processed as previously explained [48]. Samples were then subjected to SDS/PAGE (10% gels), transferred to nitrocellulose membranes and immunostained with primary antibodies to: PARP (Cell Signaling Technology Inc.), Lyn (Santa Cruz), Lyn-Tyr396 (Epitomics Onc.; Burlingame, CA), ERK (Cell Signaling Technology Inc.), ERK-Thr202/Tyr204 (Cell Signaling Technology Inc.), β-actin (Sigma-Aldrich), using an enhanced chemiluminescent detection system (Pierce; Rockford, IL).

### Flow cytometry analysis

Freshly isolated CLL B cells were cultured with and without MSCs and treated with 5μM kinase inhibitors as above described. Cells (5×10^5^ for each assay) were collected after 48h, leaving intact the adherent layer, and stained with anti-CD49d PE (BD Biosciences), anti-CCR7 FITC (R&D Systems Inc., Minneapolis, MN, USA), anti-CXCR4 PE (R&D Systems Inc.) anti-CXCR3 PE-Cy5.5 (BD Biosciences, Franklin Lakes, NJ, USA), anti-CXCR5 FITC (R&D Systems Inc.), and anti-CD19 APC (BD Biosciences) monoclonal antibodies. Cells were washed with PBS1X and incubated with saturating concentrations of the appropriate antibodies for 15 minutes at room temperature. 20,000 total events were acquired using FACSCanto A (Becton Dickinson) and the data were analysed by FACSDiva 7 software. Samples were gated on intact cells by forward scatter (FSC) *vs* side scatter (SSC). For analysis, a second gating step on CD19+ cells was used. Here, we used a difference between the Mean Fluorescence Intensity (MFI) of fully-stained samples and the Fluorescence Minus One (FMO) controls.

### Chemotaxis assay

To obtain the MSC-conditioned medium (MSC-CM), MSCs were cultured for 48h in complete RPMI-1640 medium (Sigma-Aldrich) at 37°C in a humidified atmosphere containing 5% CO_2_. The migration of pretreated CLL B cells in response to MSC-CM was evaluated using 12-well Corning chemotaxis chamber (Corning Life Sciences; Acton, MA). Briefly, 2.5×10^6^ cells were incubated in 0.5ml RPMI medium with and without kinase inhibitors for 1h at 37°C. Then, cells were transferred into the top chambers of transwell culture insert with a pore size of 3μm. Filters were then placed onto wells containing complete RPMI medium or MSC-CM, and CLL B cells were allowed to migrate for 3h at 37°C. Migrated cells in the lower chamber were then collected and counted on a FACSCanto A for 60 seconds in triplicates.

### Migration assay evaluating CLL pseudoemperipolesis

Pseudoemperipolesis is an *in vitro* phenomenon in which CLL cells spontaneously migrate beneath marrow stromal cells to mimic the *in vivo* migration and homing to stromal cells in the tissues [50]. Briefly, MSCs were seeded onto 12-well plates in complete RPMI medium till confluence. CLL B cells were suspended to a concentration of 2×10^6^ cells/ml in complete RPMI medium with or without 5μM Bafetinib, CAL-101 and Ibrutinib and incubated for 1h at 37°C in 5% CO_2_ in complete RPMI medium. After incubation, CLL cells were added to the MSC layers and the plates were incubated at 37°C in 5% CO_2_ overnight. Cells that had not migrated into the stromal cell layer were removed by vigorously washing 3 times with RPMI 1640 medium. The complete removal of non-migrated cells and the integrity of the stromal cell layer containing transmigrated cells were assessed by phase-contrast microscopy and documented photographically. The stromal cell layer containing transmigrated cells was detached by incubation with Accutase (Sigma-Aldrich). Cells were stained with anti-CD19 APC to exclude MSCs and counted by flow cytometry.

### Statistical analysis

Statistical analysis was performed using Student's *t* test, Fisher's exact test and ANOVA test. Data are reported as median ± standard deviation (SD) and a p-value of 0.05 or less was considered as significant.

## SUPPLEMENTARY MATERIAL FIGURES


