# Supplementary material for: Cross-talk between chronic lymphocytic leukemia (CLL) tumor B cells and mesenchymal stromal cells (MSCs): implications for neoplastic cell survival
Source: Oncotarget. 2015 Oct 26;6(39):42130–49. doi: 10.18632/oncotarget.6239 (PMC4747215; doi:10.18632/oncotarget.6239)
Supplement: Supplementary file 1 [file oncotarget-06-42130-s001.pdf]

# Cross-talk between chronic lymphocytic leukemia (CLL) tumor B cells and mesenchymal stem cell (MSC): implications for neoplastic cell survival

## Supplementary Material

### Characterization of MSCs obtained from CLL patients

MSCs phenotype was evaluated at early passages ($P_2$-$P_3$) by the expression of markers CD14, CD31, CD34, CD73 (Becton Dickinson; Franklin Lakes, USA), CD90 (Abd Serotech; Oxford, UK), CD45 and CD105 (Caltag-Invitrogen; Paisley, UK). Briefly, cells were washed with PBS1X, incubated with saturating concentrations of the appropriate antibodies for 15 minutes at room temperature and analyzed using FACSCalibur (Becton Dickinson) and CellQuestPRO analysis software. The capability of selected MSCs to differentiate into mesenchymal lineages (i.e. adipocytes and osteocytes) was also assessed. For adipogenic differentiation, α-MEM medium was supplemented as follows: 0.2% Indomethacin, 0.1% Isobutilmetilxantin, 1.1% Recombinant human Insulin, 0.5% Dexamethasone, 11% Mesenchymal Cell Growth Supplement (MCGS) and 2% L-glutamin (Adipogenic Induction medium; Cambrex Bio Science; Walkersville, USA); for the adipogenic maintenance, α-MEM medium was supplemented with 1.1% Insulin, 2% L-glutamin and 11% MCGS (Adipogenic Maintenance medium; Cambrex Bio Science). For optimal differentiation, induction medium was replaced every 3 days, by maintenance medium for three cycles. Lipid vacuoles were observed 3 weeks later. Once obtained, adipogenic cultures were washed with PBS1X, fixed with 4% formaldehyde (Immunotech; Marseille, France) for 10 minutes at room temperature and then stained with Oil Red O solution (Sigma-Aldrich). RNA samples were isolated and RT-PCR was performed for the adipogenic marker gene peroxisome proliferator-activated receptor γ2 (PPAR-γ2). Osteogenic differentiation of MSCs was performed by incubating the cells in osteogenic differentiation medium with 1% β-Glycerophosphate, 0.5% Dexamethasone, 0.5% Ascorbic Acid, 11% Mesenchymal Cell Growth Supplement (MCGS) and 2% L-glutamin up to 4 weeks (Cambrex Bio Science). After 2 weeks, mineralization was assessed by Von Kossa staining. RNA samples were isolated and investigated by RT-PCR for the osteogenic marker gene Core Binding Factor 1 (CBFA1).

### RT-PCR for MSC adipogenic and osteogenic induction

Total RNA from differentiated MSCs was extracted using RNeasy Mini Kit Protocol (Qiagen; Hilden, Germany) and Reverse Transcription-PCR (RT-PCR) was performed according to the manufacturer's protocol of SuperScript III First-Strand kit (Life Technologies; Paisley, UK). RT-PCR amplifications were performed using specific primers for Peroxisome Proliferator Activated Receptor γ2 (PPARγ2) [forward primer: 5'-TGA ACG ACC AAG TAA CTC TCC-3', reverse primer: 5'-CTC ATG TCT TGT CTC TCC GTC TTC-3'] and Core Binding Factor α1 (CBFA1) [forward primer: 5'-GGC CTT CCA CTC TCA GTA AGA-3', reverse primer: 5'-GAT TCA CCA TTC TGC CAC TA-3'] on GeneAmp PCR System 2700 (Applied Biosystem; Foster City, USA). PCR reactions were performed under the following conditions: initial denaturation at 95°C for 40 seconds, followed by 62°C ($T_{an}$ of PPARγ2) or 55°C ($T_{an}$ of CBFA1) for 40 seconds, 72°C for 40 seconds cycled 35 times and 72°C for 7 minutes.

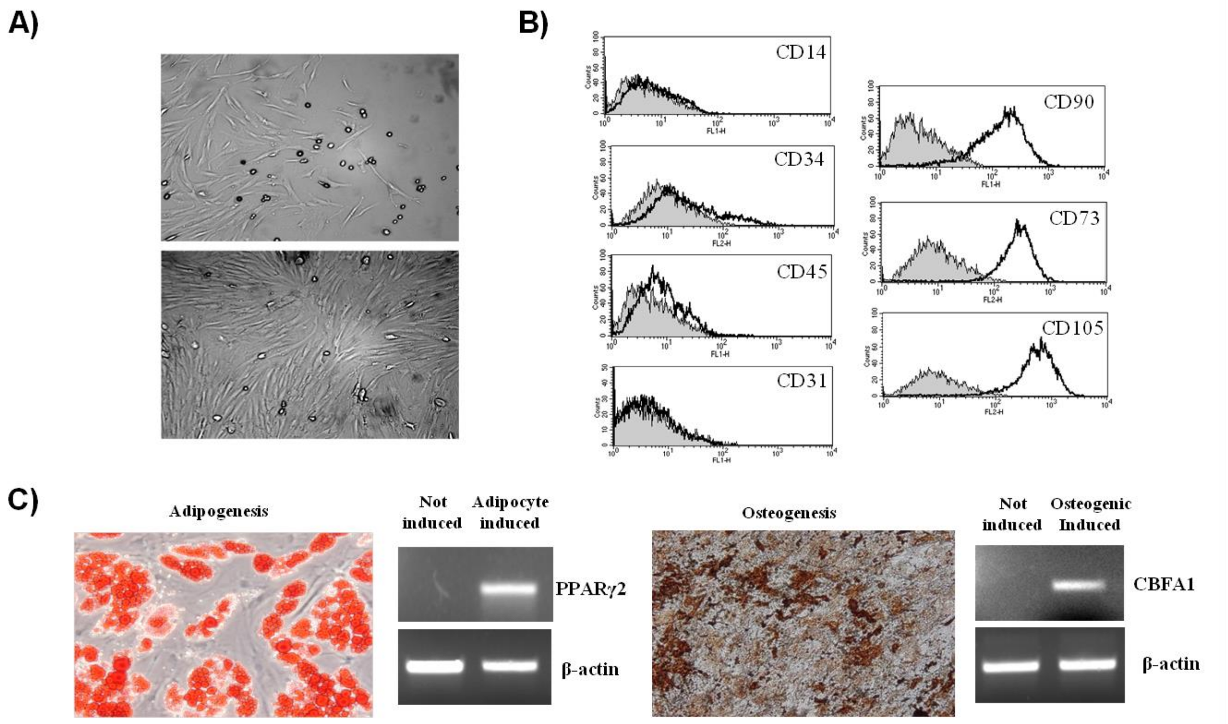


**Figure S1. Characterization of MSCs cultures from CLL patients. A)** Once attached, cells with a fibroblastic-like morphology leads to the formation of high proliferating colony, achieving the confluence after 30 days (10X magnification, Olympus BX60). **B)** A representative case of MSC immunocharacterization. **C)** MSC differentiation into mesenchymal lineages: adipocytes and osteocytes induction was evaluated with specific stainings (Oil Red staining for adipocytes and Von Kossa staining for osteoblasts) (10X magnification, Olympus IX81) and molecular analysis with specific primers for the following genes: PPAR-γ (Peroxisome Proliferator Activated Receptor) and CBFA1 (Core Binding Factor 1).

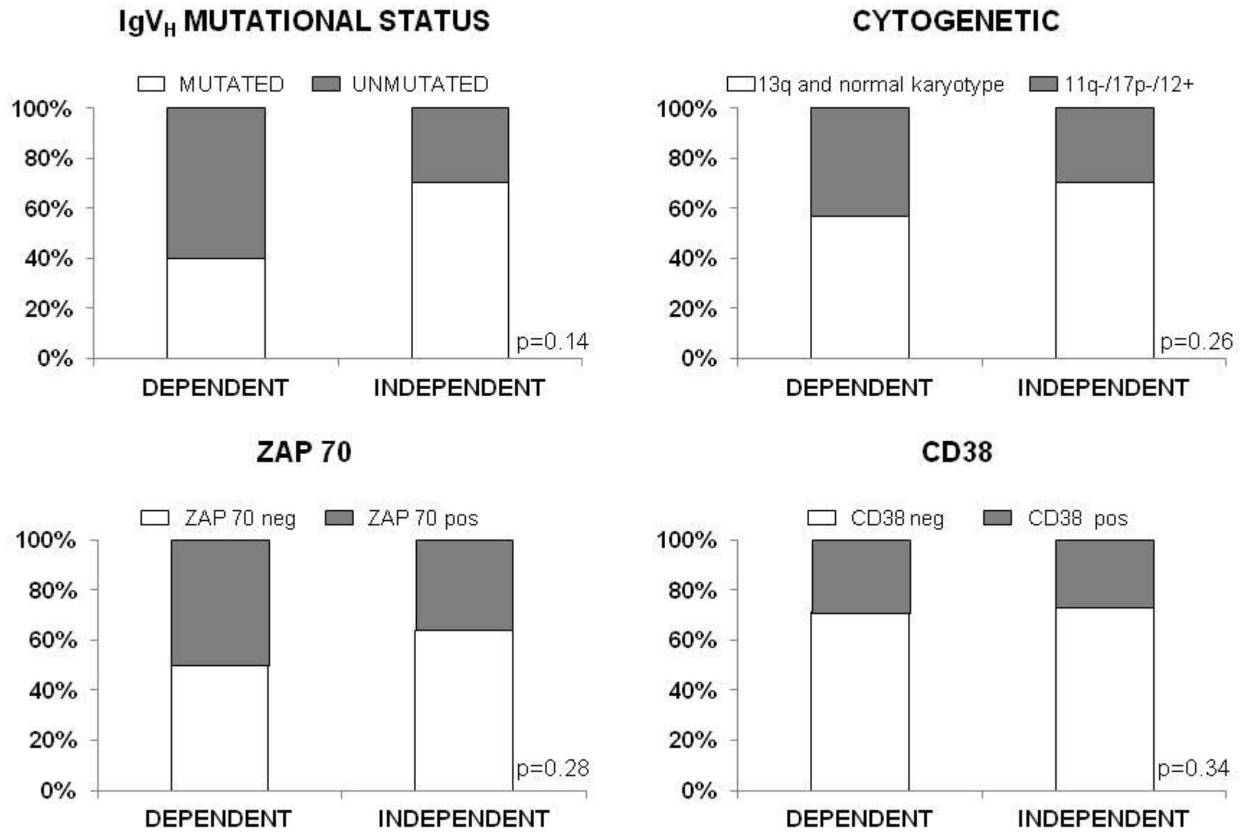


**Figure S2. Correlation between PARP cleavage pattern and CLL prognostic markers**. Data obtained from the western blotting analysis of the PARP protein were related to the most common CLL prognostic markers: Ig$V_H$ mutational status, cytogenetic, ZAP70 and CD38 expression. No correlation was found in any case. Fisher's exact test.

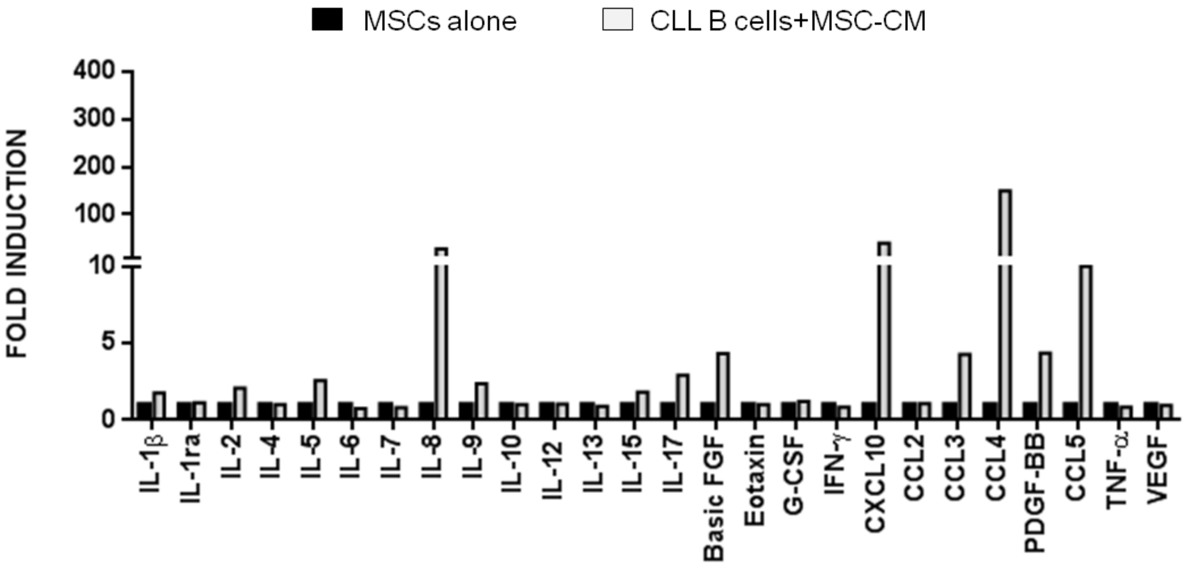


**Figure S3. Cytokine/Chemokine secretion profile of CLL B cells in presence of MSC conditioned medium.** CLL B cells were cultured in medium alone or in presence of MSC-CM for 7 days and the level of cytokines in supernatants was assessed by Bio-Plex (n=7). Data are expressed as Fold Induction (FI) of cytokines/chemokines quantification in supernatants of CLL B cells+MSC-CM normalized on MSCs alone.

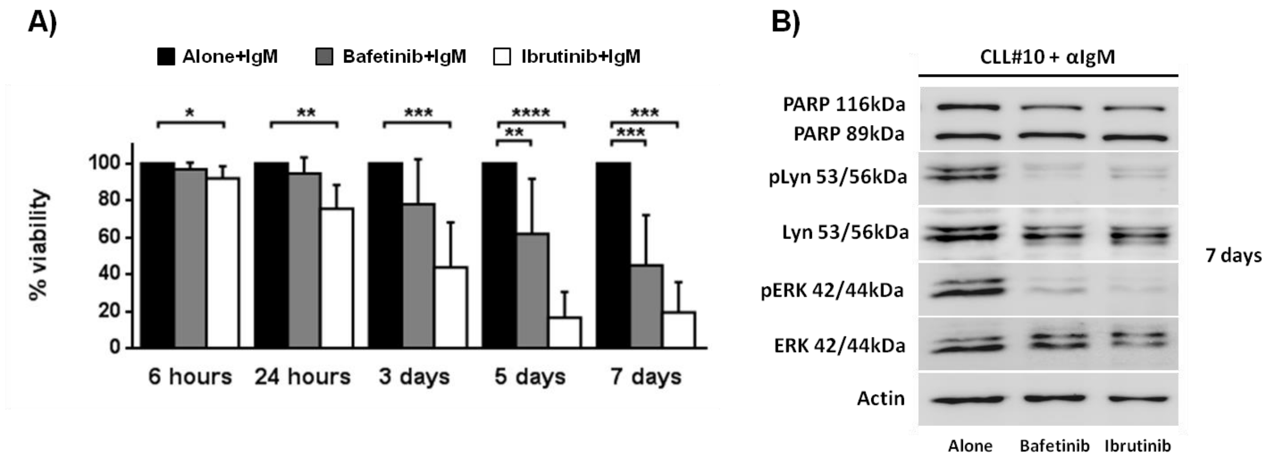


**Figure S4. Kinase inhibitors counteract the pro-survival effect mediated by BCR stimulation.** CLL B cells were pre-treated with 5μM Bafetinib and 5μM Ibrutinib before the addition of anti-IgM. **A)** CLL B cells were cultured for 6 hours, 24 hours, 3, 5 and 7 days; the graphs show the mean ± standard deviation of Annexin $V^{neg}$ cells percentage of 9 separated experiments compared with untreated controls; Student's paired *t* test; *$p<0.05$, **$p<0.01$, ***$p<0.001$ and ****$p<0.0001$. **B)** The total cell lysates were subjected to SDS-PAGE, transferred to nitrocellulose membrane and detected sequentially with: anti-PARP, anti-Lyn-Tyr396, anti-Lyn, anti-ERK-Thr202/Tyr204, anti-ERK and anti-β-actin. The figure shows a representative case of CLL B cells (CLL#) treated with 5μM Bafetinib and 5μM Ibrutinib and stimulated with anti-IgM (10μg/ml).

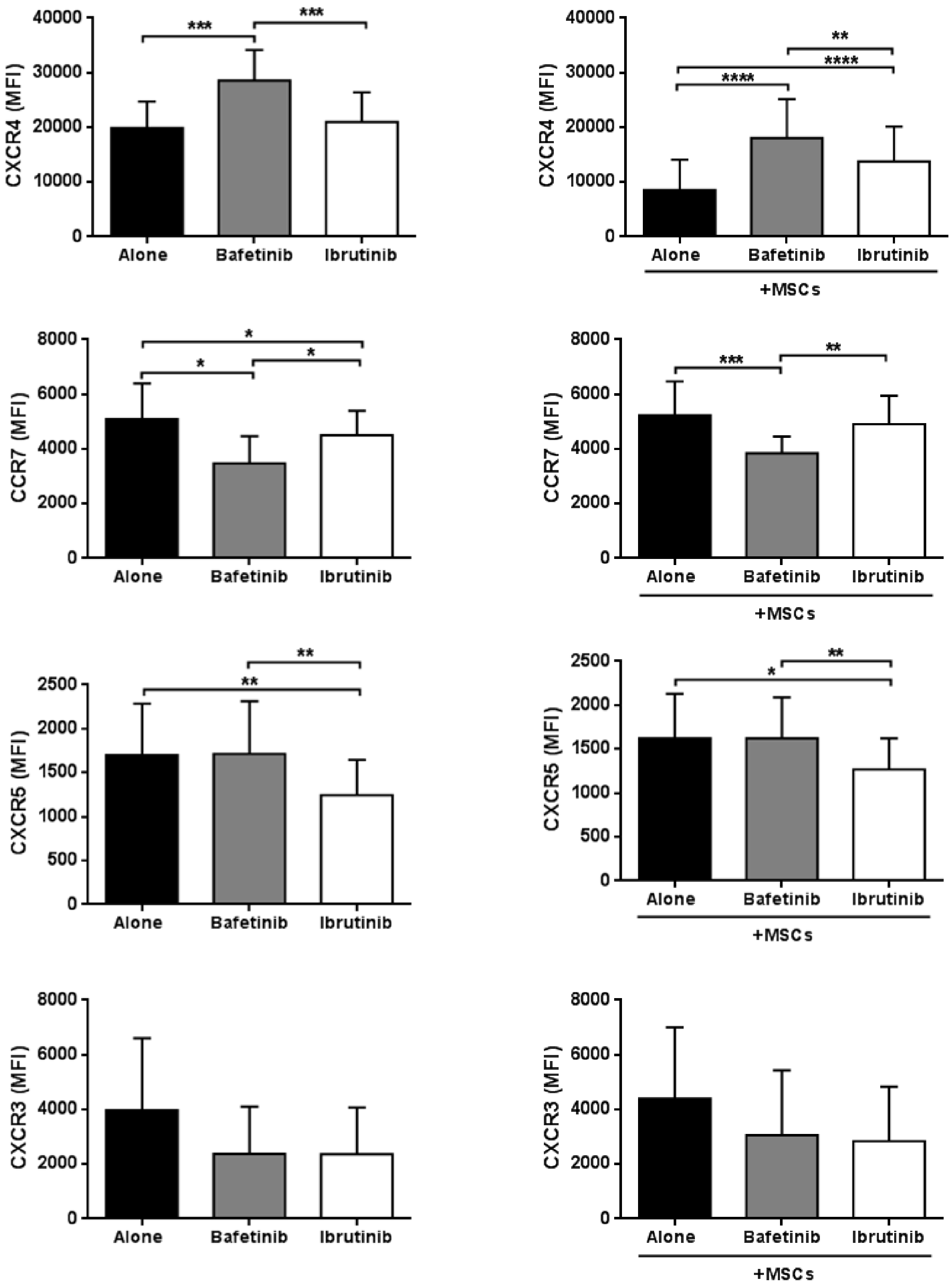


**Figure S5. Kinase inhibitors effect on chemokine receptor surface expression.** Leukemic B cells (n=11) treated with 5µM Bafetinib and Ibrutinib were cultured alone or with MSCs for 48h and the expression level of CXCR4, CCR7, CXCR5 and CXCR3 was assessed by flow cytometry. Data are presented as mean ± standard deviation of MFI for each receptor; paired Student's *t* test; *p<0.05, **p<0.01, ***p<0.001 and ****p<0.0001 with respect to untreated cells.
